# Detection of Metabolic Fluxes of O and H Atoms into Intracellular Water in Mammalian Cells

**DOI:** 10.1371/journal.pone.0039685

**Published:** 2012-07-25

**Authors:** Helen W. Kreuzer, Luca Quaroni, David W. Podlesak, Theodora Zlateva, Nikki Bollinger, Aaron McAllister, Michael J. Lott, Eric L. Hegg

**Affiliations:** 1 Pacific Northwest National Laboratory, Richland, Washington, United States of America; 2 Paul Scherrer Institut, Villigen, Switzerland; 3 Department of Biology, University of Utah, Salt Lake City, Utah, United States of America; 4 Saskatchewan Cancer Research Unit and Department of Biochemistry, University of Saskatchewan, Saskatoon, Canada; 5 Department of Chemistry, University of Utah, Salt Lake City, Utah, United States of America,; 6 Department of Biochemistry & Molecular Biology, Michigan State University, East Lansing, Michigan, United States of America; Université Joseph Fourier, France

## Abstract

Metabolic processes result in the release and exchange of H and O atoms from organic material as well as some inorganic salts and gases. These fluxes of H and O atoms into intracellular water result in an isotopic gradient that can be measured experimentally. Using isotope ratio mass spectroscopy, we revealed that slightly over 50% of the H and O atoms in the intracellular water of exponentially-growing cultured Rat-1 fibroblasts were isotopically distinct from growth medium water. We then employed infrared spectromicroscopy to detect in real time the flux of H atoms in these same cells. Importantly, both of these techniques indicate that the H and O fluxes are dependent on metabolic processes; cells that are in lag phase or are quiescent exhibit a much smaller flux. In addition, water extracted from the muscle tissue of rats contained a population of H and O atoms that were isotopically distinct from body water, consistent with the results obtained using the cultured Rat-1 fibroblasts. Together these data demonstrate that metabolic processes produce fluxes of H and O atoms into intracellular water, and that these fluxes can be detected and measured in both cultured mammalian cells and in mammalian tissue.

## Introduction

Metabolic processes in heterotrophs introduce H and O atoms into water via a variety of processes. For example, O atoms in CO_2_ [1] and loosely held H atoms such as those in hydroxyl groups [2] rapidly exchange with O and H atoms in intracellular water. Similarly, carbonyl O atoms undergo uncatalyzed exchange with water via hydration and dehydration, and H atoms bound to a carbon adjacent to a ketone exchange with water via keto-enol tautomerism [3], [4]. Both O and H atoms can also enter intracellular water via dehydration reactions (e.g. the dehydration of 2-phospho-glycerate during glycolysis or the condensation reactions involved in protein and nucleic acid biosynthesis). Finally, substrate H atoms donated to FAD or NAD^+^ during enzymatic reactions are released during electron transport, when oxygen atoms from atmospheric O_2_ are also incorporated into intracellular water. Both H and O atoms have been traced from substrates into water *in vitro* by direct NMR measurements [5], [6], and kinetics of dilution and elimination of ^2^H and ^18^O from doubly labeled water is a standard method to measure total body metabolic rate [7].

Despite the universal recognition of O and H fluxes as part of metabolic activity, those fluxes remain uncharacterized in mammalian cells. Kreuzer et al. [8]–[10] recently used isotope ratio mass spectrometry (IRMS) to demonstrate that fluxes of O and H into intracellular water of *E. coli* could be detected and quantified. In exponentially-growing cells, the flux of O from metabolic activity comprised approximately 70% of the O atoms in intracellular water, while the H flux comprised approximately 50% of those atoms. In stationary phase cells, the fluxes diminished to approximately 30%. These studies not only revealed the presence of O and H fluxes from metabolic activity in intracellular water, they also established that the H fluxes could be traced into cellular lipids [10]. Thus, the isotopic disequilibrium across the plasma membrane of this gram-negative bacterium is maintained long enough for metabolic fluxes of O and H atoms in intracellular water to be reproducibly detected. Very recently, analysis of H isotope ratios of lipids from cyanobacteria [11] and dinoflagellates [12] as a function of growth water salinity provided data that was consistent with a measurable isotopic gradient across the membrane in these organisms as well.

In this paper, we report the detection of metabolic fluxes of O and H atoms in mammalian cells. We apply the mass spectrometric approach of Kreuzer et al. [8]–[10] to detect these fluxes specifically in the intracellular water. We then employ a novel orthogonal infrared spectromicroscopy approach [13], [14] to detect *in real time* the flux of H atoms in cultured rat cells associated with metabolic activity. Finally, we demonstrate that water extracted from rat muscle tissue contains O and H atoms that are isotopically distinct from those in total body water, as would be expected from metabolic fluxes *in vivo*.

## Results

### Detection of Isotopically Distinct Water in Cultured Fibroblasts by IRMS

Four sets of Rat-1 fibroblasts were grown in Dulbecco Modified Eagle Medium (DMEM) +10% calf serum made with four isotopically distinct batches of water, which were made by spiking laboratory water with varying amounts of ^2^H_2_^18^O. The cells were harvested by gravity filtration either during exponential growth (30% confluence) or during their quiescent phase (100% confluence) and immediately frozen. Water was then extracted cryogenically from the cell pellet as previously described [9]. This experiment was conducted twice for 30% confluent cells and twice for 100% confluent cells.

The extracted water consists of both intracellular and extracellular water. If it contains fluxes of O and H atoms from metabolic activity, presumably with an isotopic content distinct from that of growth medium water, then the O and H isotopic composition of the extracted water will not be identical to that of growth medium water. The O or H composition of the extracted water (EW) can be modeled simply as a two-component mixture where
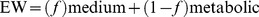
(1)and its isotope ratio modeled accordingly:
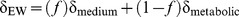
(2)where *f* is the fraction of O or H atoms in extracted water that is isotopically equivalent to growth medium water, (1 - *f*) is the flux of O or H atoms in extracted water presumed to be derived from metabolic activity, δ^medium^ is the isotope ratio of the growth medium water, and δ^medium^ is the isotopic content of the metabolic flux. Equations 1 and 2 represent a straight line in the form of *y*  =  m*x* + b.

**Figure 1:**
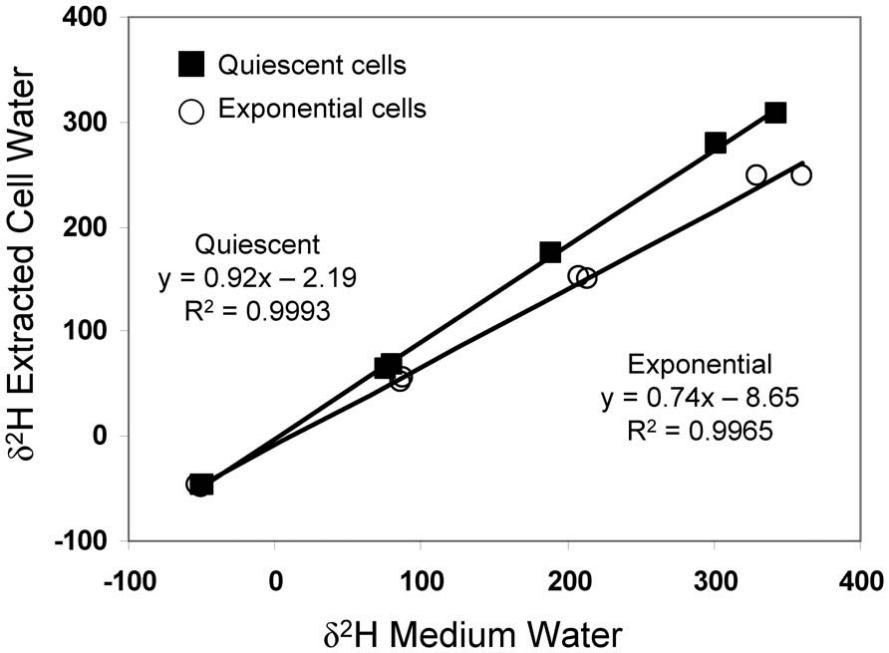
Regression of the hydrogen isotope ratio of extracted water versus growth medium water. Rat-1 fibroblasts grown in DMEM were harvested either during exponential growth (30% confluent) or after they reached quiescence (100% confluent). 26% (or 1– slope) of the H atoms in the total water extracted from the cell cakes was isotopically distinct from growth medium water when the cells were in exponential phase. This value dropped to 8% when the cells reached quiescence. Similar values are obtained with oxygen isotopes, and all of these results are summarized in Table 1.

In Figure 1 we plot the H isotope ratios of extracted water versus those of the culture medium water for 30% confluent cells (exponentially growing) and 100% confluent (quiescent) cells. The regression statistics for both the O and H isotope analyses are presented in Table 1. These data indicate that ∼26% (1 - *f*) of the H atoms in the water extracted from exponentially growing cells were isotopically distinct from the culture medium water. In the quiescent cells, that proportion dropped to ∼8%. Thus, in quiescent cells, which are presumably less metabolically active than cells undergoing exponential growth, the proportion of isotopically distinct H atoms was less. Similar patterns were seen for the O atoms, where ∼27% of the population was isotopically distinct from growth medium water in exponentially-growing cells, while only ∼11% of the population of O atoms extracted from quiescent cells was isotopically distinct from the culture medium water. These data are consistent with the explanation that isotopically distinct O and H arise from metabolic activity.

**Table 1:** Regression coefficients of tissue culture “growth” and “wash” experiments, and estimations of metabolic flux.

Experiment	H isotope data				O isotope data			
	Regression coefficient	Standard error	Intercept	Standard error	Regression coefficient	Standard error	Intercept	Standard error
Growth experiments to determine (*f)* inexponentially growing cells	0.74	0.018	−8.6	3.8	0.73	0.038	2.1	0.5
Growth experiment to determine (*f)* in quiescent cells	0.92	0.011	−2.2	2.1	0.89	0.012	1.6	0.1
Washing to determine (*g)* in exponentially growing cells	0.53	0.025			0.54	0.29		
Washing to determine (*g)* in quiescent cells	0.62	0.024			0.63	0.23		
Estimated flux (% atoms from metabolism in intracellular water) in 30% confluent cells	0.55				0.59			
Estimated flux (% atoms from metabolism in intracellular water) in quiescent cells	0.21				0.30			

Using Equation 2, we can also calculate estimated isotope ratios for the isotopically distinct populations, where δ_metabolic_  =  *y*-intercept/(1– *f*). The H isotope ratios for the isotopically distinct populations in 30% confluent and 100% confluent cells are approximately −33‰ and −28‰, respectively, while the O isotope ratios for the isotopically distinct populations are approximately +8‰ and +15‰.

### Real-time Detection of Fluxes of H from Cultured Fibroblasts by Fourier Transform Infrared (FTIR) Spectromicroscopy

To gain further evidence that the fluxes of isotopically distinct atoms seen in water extracted from cultured cells were derived from metabolic activity, we employed a novel FTIR spectromicroscopy method that enables separate detection of absorption by ^1^H_2_O, ^1^H^2^HO, and ^2^H_2_O at high spatial resolution. For these experiments, Rat-1 fibroblasts were cultured directly on CaF_2_ optical windows until the cells reached 60% confluence. The cells were then washed two times with serum-free DMEM medium prepared in 99% ^2^H_2_O (which replaced all the extracellular medium water with ^2^H_2_O), covered with a thin film of ^2^H_2_O-prepared DMEM medium, and analyzed via FTIR spectroscopy over the course of several hours.

**Figure 2:**
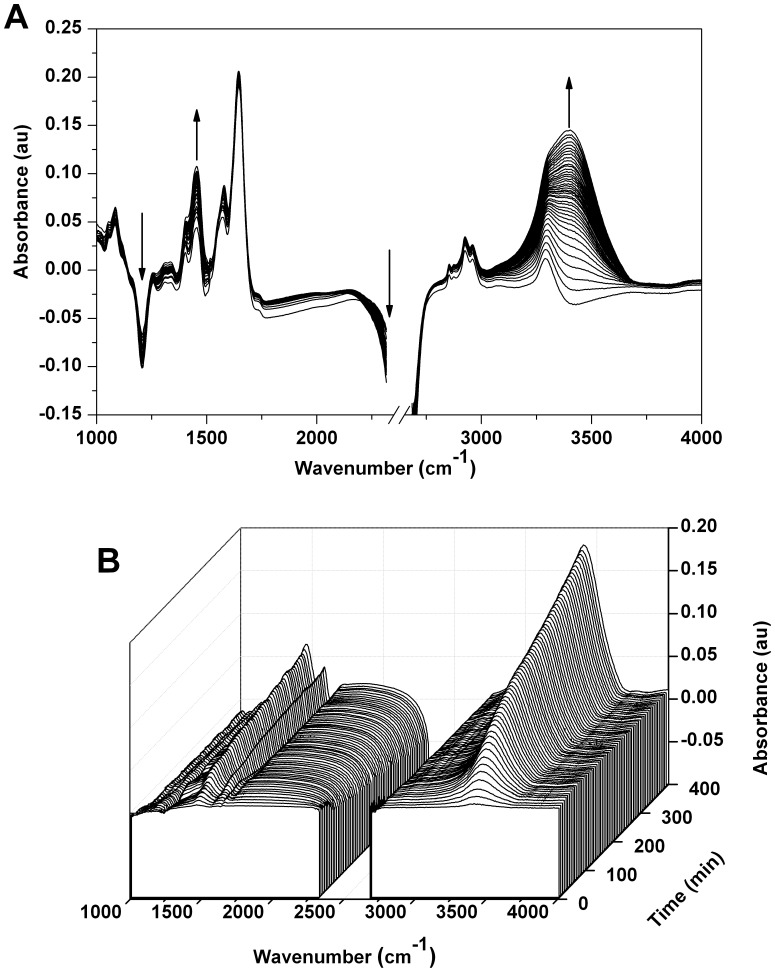
FTIR spectromicroscopy difference spectra of Rat-1 Fibroblasts. FTIR spectra from a cluster of Rat-1 cells grown on a CaF_2_ optical slide (∼60% coverage). Spectra were recorded (25×25 µm^2^ aperture) over 360 minutes after washing the cells with DMEM prepared with 100% ^2^H_2_O. *A*, a two-dimensional plot depicting the absolute absorbance spectra after subtracting the background (an area with ^2^H-O-^2^H but no cells). *B*, the same data plotted in three dimensions in which the initial spectrum (defined as t  = 0) of a cell cluster was used as the reference. In both *A* and *B* the spectra are recorded every 5 minutes. The bands at ∼3400 cm^−1^ (^1^H-O-^2^H stretch) and ∼1450 cm^−1^ (^1^H-O-^2^H bend) increase over time while the bands at ∼2300 cm^−1^ (shoulder of the ^2^H-O-^2^H stretching mode) and ∼1225 cm^−1^ (^2^H-O-^2^H bend) decrease over time.

Figure 2 displays IR spectra over time of a cell cluster that essentially filled the optical aperture such that the water being detected was either inside the cells or in close proximity to them. The spectra in Figure 2*A* have been plotted as absolute absorbance spectra after subtracting the background (an area with ^2^H-O-^2^H but no cells); negative “peaks” indicate that there is more ^2^H-O-^2^H in the background than in the cell cluster. In addition, this same data has also been plotted as differential absorbance variations (Figure 2*B*) in which the spectra were normalized by subtracting the first IR spectrum (defined as t  = 0) of a cell cluster from subsequent spectra of the same cluster, so that decreasing signals form negative “peaks” while increasing signals form positive peaks. Thus, Figure 2*A* exhibits absorption bands from all the molecular components of the cells whereas Figure 2*B* only reveals spectral contributions from those components that are changing over time. In both Figure 2*A and 2B*, the absorption of the band from the ^2^H-O-^2^H stretch at ∼2600 cm^−1^ is saturated, but the shoulder at 2300 cm^−1^ can still be used to follow the change in concentration of ^2^H-O-^2^H.

The spectra in Figure 2*A* are dominated by absorption bands characteristic of cellular components. In particular, the bands at 1545 cm^−1^ and 1650 cm^−1^ (Amide I and Amide II, respectively) are mostly due to absorption from protein peptide bonds, while the band at 3280 cm^−1^ (Amide A) results from the stretching vibration of the peptidic N-H bond. Other major bands that are characteristic of abundant biomolecules include bands in the 2800–3000 cm^−1^ region (lipid acyl chain vibrations) as well as those in the 1000–1300 cm^−1^ region (polysaccharide and phosphate ester C-O and P-O vibrations).

It should be noted that at the onset of the measurement, a few minutes after exposing the cells to medium prepared with 99% ^2^H_2_O, both the Amide II and Amide A band are clearly present in the spectrum, albeit with somewhat reduced intensity. (The Amide II band is roughly half the intensity of the Amide I band in typical cellular spectra, whereas it appears to be only 30% of the Amide I in Figure 2*A*.) This indicates that only limited exchange of the peptidic ^1^H atoms with ^2^H atoms has occurred inside the cell (exchange of peptide ^1^H atoms with ^2^H leads to a shift to lower wavenumbers). Proteins in solution normally exchange solvent exposed peptidic ^1^H atoms within seconds in the presence of a solution of ^2^H_2_O, although internal ^1^H atoms that are excluded from the solvent often require minutes to hours for exchange. Our observation of limited ^1^H/^2^H exchange inside the cell suggests that intracellular water has not fully equilibrated with the extracellular water and supports the suggestion that an isotopic concentration gradient exists across the cell membrane. Significantly, the persistence of the Amide II and Amide A bands throughout the course of the six hour experiment (Figure 2*A*) strongly suggests that this gradient is maintained over time.

Figure 2*B* highlights the changes in spectral absorption occurring over the course of the experiment. The largest changes are an increase of the bands at ∼3400 cm^−1^ and ∼1450 cm^−1^ resulting from the ^1^H-O-^2^H stretching and bending modes, respectively, and a decrease of the bands at ∼2600 cm^−1^ (represented by the change of the side of the band at 2300 cm^−1^) and ∼1225 cm^−1^ due to the corresponding ^2^H-O-^2^H modes. The significant increases over time in the ^1^H-O-^2^H signals as well as the decreases in the ^2^H-O-^2^H signals are consistent with light atoms replacing heavy atoms over the course of the experiment. Interestingly, comparable FTIR spectral changes were also observed when the unicellular green alga *Chlamydomonas reinhardtii* was incubated in growth medium prepared with 80% ^2^H_2_O [14], although these changes were not explicitly discussed.

What could be the source of the ^1^H-O-^2^H detected in the FTIR spectra? Although the culture medium water was replaced by ^2^H_2_O, the cells themselves presumably still contained a distinct fraction of ^1^H_2_O in their intracellular water, a notion supported by the substantial intensity of the Amide II and Amide A bands indicating peptidic ^1^H atoms. Thus, one source of the ^1^H-O-^2^H could be ^1^H/^2^H exchange after ^1^H diffused passively across the cell membrane and mixed with extracellular ^2^H_2_O molecules. Another possibility is that H exchange from loosely held atoms on organic molecules (such as N-H groups) introduced ^1^H into the water. However, no significant change was observed in the absorbance band at 3280 cm^−1^ (Amide A), suggesting that the isotopic composition of intracellular water is close to a steady state over the course of the experiment. Finally, metabolic activity of the cells could be introducing a flux of ^1^H into the system. Although the cells were rinsed with DMEM made with 100% ^2^H_2_O, the nutrients in the medium were not deuterated and would therefore contain primarily ^1^H. Metabolic processing of these nutrients would release ^1^H into intracellular water, from whence it could diffuse into extracellular water.

**Figure 3:**
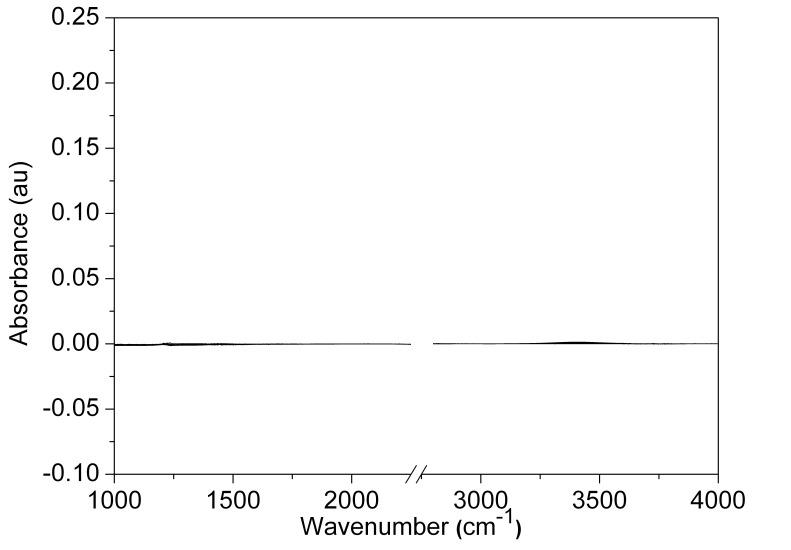
FTIR spectromicroscopy difference spectra of Rat-1 Fibroblasts in lag phase. FTIR spectra from a cluster of lag phase Rat-1 cells grown on a CaF_2_ optical slide. Spectra were recorded (25×25 µm^2^ aperture) over 160 minutes after washing the cells with DMEM prepared with 100% ^2^H_2_O The initial spectrum (defined as t  = 0) was used as the reference. The scale is the same as that used in Figure 2*A*. The horizontal line indicates that there is essentially no difference between the various spectra.

To determine if the production of ^1^H-O-^2^H was dependent on the metabolic state of the cells, we performed a similar set of experiments using lag-phase cells (i.e. cells that are growing at a very slow rate after seeding as they are acclimating to their new environment). Rat-1 fibroblasts were seeded onto the CaF_2_ optical window and washed with serum-free DMEM medium prepared with ^2^H_2_O after the cells attached to the surface (4–8 hours after seeding). As in Figure 2*B*, the first IR spectrum (t  = 0) of a cell cluster was subtracted from subsequent spectra of the same cluster, and the difference spectra were plotted on a single graph (Figure 3). The resulting horizontal line in the graph reveals that there are minimal changes in the absorbance of either the ^1^H-O-^2^H or ^2^H-O-^2^H bending and stretching modes during the course of the experiment (160 min) when less metabolically active lag-phase cells are utilized.

**Figure 4:**
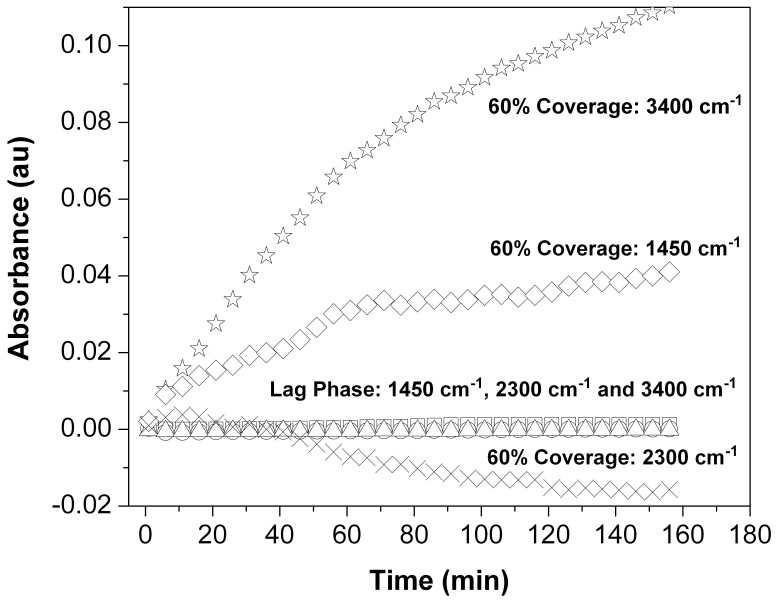
Plot of change in intensity over time of the absorption bands due to H_2_O stretching and bending modes. FTIR spectromicroscopy difference spectra recorded from a sample of lag phase cells (Figure 3) and a sample of cells at 60% coverage (Fig. 2) bathed in DMEM prepared with 100% ^2^H_2_O. The first spectrum taken (defined as t  = 0) is the reference spectrum. 3400 cm^−1^ = ^1^H-O-^2^H stretch; 1450 cm^−1^ = ^1^H-O-^2^H bend; 2300 cm^−1^ =  shoulder of ^2^H-O-^2^H stretch; (⋆) 60% Coverage Cells: 3450 cm^-1^; (□) Lag Phase Cells: 3450 cm^-1^; (⋄) 60% Coverage Cells: 1450 cm^−1^; (○) Lag Phase Cells: 1450 cm^−1^; (×) 60% Coverage Cells: 2300 cm^−1^; (▵) Lag Phase Cells: 2300 cm^−1^. For the cells at 60% coverage, the absorptions due to ^1^H-O-^2^H increase over time while the absorption due to ^2^H-O-^2^H decreases. Those same absorptions in lag phase cells show essentially no change over the course of this experiment.

The sensitivity of the IR spectra to the metabolic state of the Rat-1 fibroblasts is further illustrated in Figure 4. In the spectra of the metabolically active cells, the shoulder of the ^2^H-O-^2^H stretching mode (2300 cm^−1^) decreases over time while the peaks due to the ^1^H-O-^2^H stretch (3400 cm^−1^) and bend (1450 cm^−1^) increase, illustrating the flux of ^1^H entering the water pool via metabolic processes. Conversely, in the less metabolically active lag-phase cells, the intensities of those same peaks do not change appreciably over time. This supports the conclusion that at least some of the isotopically distinct H and O atoms detected in water extracted from fibroblasts represent this same metabolic flux.

### Intracellular vs. Extracellular Water

While the FTIR experiments clearly demonstrate that the flux of ^1^H into the entire system requires metabolic activity, they cannot reveal the magnitude of the intracellular metabolic flux. Kreuzer et al. [8]–[10] previously developed an approach to calculate the amount of intracellular versus extracellular water in a cell sample using isotope ratio mass spectrometry, and we employed this method on the Rat-1 fibroblasts.

Samples of Rat-1 fibroblasts harvested on filters contain both intracellular and extracellular water. Thus, water extracted from those samples is a mixture of both intra- and extracellular water, and can be described as a two-component mixture:
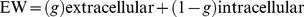
(3)where EW  =  extracted water, *g* is the fraction of that water composed of extracellular water, and (1 - *g*) is the fraction of the extracted water composed of intracellular water.

The isotopic content of the extracted water can be modeled equally simply, using either R or delta values:
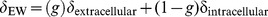
(4)where δ is used to signify the isotope ratio of the various components.

To determine the values of *g* and (1 - *g*), a batch of cultured cells was split into four portions and filtered. After the growth medium had filtered away from the cells, each portion was briefly washed with fresh growth medium made in isotopically distinct water. The washing step replaced the extracellular water with water of known isotopic content. This experiment was performed with exponentially growing (∼30% confluent) and quiescent (∼100% confluent) cells. Regression of the isotopic content of the extracted water upon the known values of the wash solution yielded a regression coefficient equal to *g*, and (1 - *g*) is equal to the fraction of the extracted water that is intracellular water.

The regression statistics from the “washing” experiments for both O and H isotopic content in the actively growing and confluent cells are presented in Table 1. Importantly, the coefficients for both O and H atoms in the wash experiments are statistically indistinguishable, indicating that the same percentages of intracellular versus extracellular water was detected in a given experiment, no matter whether O or H isotopes were being measured. The data show that approximately 50% of the water extracted from exponentially-growing cells and 40% of the water extracted from the quiescent cells was intracellular.

### Estimation of Intracellular Metabolic Fluxes

The metabolic fluxes of O and H in the cultured cells can be estimated using the values calculated for *f* and *g* in Table 1. If we assume that the isotopically distinct O and H atoms of the cell cake water are a product of metabolism (1 - *f* from Equation 1), a reasonable assumption given our FTIR spectromicroscopy data, and we assume that after washing the cells essentially all of these isotopically distinct atoms are within intracellular water (1 - *g* from Equation 3), then the proportion of H and O atoms in intracellular water that are derived from metabolism can be simply calculated as (1 - *f*)/(1 - *g*). These calculations yield values of ∼55% and ∼59% for the H flux and O flux, respectively, in rapidly growing 30% confluent Rat-1 fibroblasts. In contrast, in quiescent cells, only ∼21% and ∼30% of the H and O atoms in intracellular water are derived from metabolic processes (Table 1). These numbers would decrease if a portion of the isotopically distinct atoms were present in the extracellular rather than intracellular water, or if some portion of the isotopically distinct atoms occurred in the extracted water for reasons other than metabolic activity. However, given the clear FTIR evidence that metabolically active cells give rise to a flux of H atoms into water molecules, it is certain that at least some of the isotopically distinct population observed by isotope ratio mass spectrometry represents a metabolic flux.

### Detection of Isotopically Distinct Atoms in Water Extracted from Rat Tissue

Because cellular metabolic activity gives rise to fluxes of O and H atoms we could detect both by isotope ratio mass spectrometry and by FTIR spectromicroscopy, we hypothesized that it might also be possible to detect metabolically-derived O and H atoms in animal tissue. In an approach that was analogous to our tissue culture experiments, we probed water extracted directly from muscle tissue for the presence of populations of O and H atoms that were distinct from the total body water of the animal.

The isotopic content of total body water is significantly influenced by the isotopic content of the animal’s drinking water and water contained within its food [15]–[19], while the isotopic content of putative metabolic fluxes would be a function of the animal’s metabolic activity, the isotopic content of the organic molecules within its food (which contain both O and H atoms) and, presumably, the isotopic content of the oxygen in air. We reasoned that animals fed isotopically distinct drinking water but identical food would have total body water that was isotopically distinct, but that the isotopic content of the metabolic O and H fluxes in these animals would be similar. Thus, water extracted from muscle tissue from these animals would contain a fraction of water that was (a) isotopically distinct from their total body water while at the same time (b) isotopically similar among the different groups drinking isotopically distinct water.

We therefore raised seven genetically identical rats on identical food, but on water that had a H isotopic content of either +348‰ or -120‰. The O isotopic content of the water was either +15.5‰ or –16.0‰. The rats were raised on these food and water regimens until they were 3 months old, at which time the animals were sacrificed and immediately dissected. The samples were then sealed in air-tight vials and frozen at −80 °C prior to analysis. Total body water was extracted from blood, while muscle water was extracted from thin (1 mm) slices of gastrocnemius muscle. The H and O isotope ratios of the extracted water are presented in Table 2.

**Table 2:** δ^2^H and δ^18^O values of muscle tissue extracted from rats grown on three different waters.

Hydrogen Isotope Data, ‰*			Oxygen Isotope Data, ‰ **		
Drinking water	Body water	Muscle tissue water	Drinking water	Body water	Muscle tissue water
+348	+241	+212	+15.5	+6.2	+7.0
+348	+250	+234	+15.5	+7.2	+7.6
+348	+240	+197	+15.5	+5.8	+5.8
+348	+247	+208	**average**	**+6.4**	**+6.8**
+348	+250	+207	−16.0	−9.6	−7.5
**average**	**+245.6**	**+211.6**	−16.0	−9.5	−7.1
−120	−83	−77	−16.0	−9.1	−6.8
−120	−86	−79	−16.0	−9.4	−6.8
**average**	**−84.5**	**−78**	**average**	**−9.4**	**−7.05**

These data are compared with the values obtained from whole body water.

* Measurement precision for H isotopes  = 2‰.

** Measurement precision for O isotopes  = 0.3‰.

The isotopic content of both total body water and muscle tissue water extracted from the rats displayed individual variation, but there were consistent trends. First, the isotopic content of total body water was not equivalent to that of drinking water. This result was expected, and is consistent with models of water flux in animals, which incorporate fluxes from both evaporation and metabolic activity [15]–[18]. Water extracted from muscle tissue of rats that were drinking water with the lower heavy isotope content of H (−120‰) or O (−16.0‰) contained more of the heavy isotope (i.e. was isotopically enriched) compared to their total body water. Conversely, water extracted from muscle tissue of rats that were drinking water with the higher ^2^H content (+348‰) contained less of the heavy isotope than did their total body water. Water extracted from muscles of rats drinking the water containing more ^18^O (+15.5‰) had approximately the same O isotopic content as body water. This data is consistent with the notion that water extracted from muscle tissue contains a population of H atoms with an isotopic content less than +240‰ and greater than −83‰, and a population of O atoms with an isotopic content fairly close to +7.0‰.

Using a two-component mixing model, we calculated both the size and isotope ratio of the isotopically distinct O and H populations in the extracted muscle tissue water:
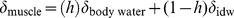
(5)where *h* is the fraction of H or O atoms in the extracted muscle water that is isotopically equivalent to total body water, (1– *h*) is the fraction of isotopically distinct water (idw), and δ_idw_ is the isotope ratio of the isotopically distinct water. To perform these calculations, we used the average values for body water and muscle tissue isotopic content for animals drinking water of the same O or H isotope ratio (Table 2). These data suggest that the proportion of isotopically distinct H atoms in the extracted muscle water was ∼12% and its isotopic content was ∼−40‰. The proportion of the isotopically distinct O atoms in the extracted water is also ∼12% with an isotopic content of ∼+10‰.

Although these data do not conclusively prove that the water extracted from the muscle tissue contained fluxes of O and H atoms derived from metabolic activity, it definitively demonstrates that there were populations of O and H atoms in the extracted muscle water that were isotopically distinct from the total body water of the animals. Further, the proportions of these isotopically distinct populations in the total extracted water were not dissimilar to those seen in water extracted from 100% confluent cultured Rat-1 fibroblasts.

## Discussion

The fluxes of H and O in a cell are a complex and poorly understood process that includes the breakdown and biosynthesis of cellular components, the catabolism and conversion of external nutrients, the reduction of O_2_, and the diffusion of H_2_O into and out of the cell. Intracellular water is the nexus of these different processes, and being able to monitor intracellular water dynamics is therefore central to understanding the fluxes of H and O in biological systems.

In this manuscript we demonstrate that a large fraction of the H and O atoms in the intracellular water of mammalian cells is isotopically distinct from extracellular water. In particular, our data reveal that slightly over 50% of both H and O atoms in intracellular water extracted from exponentially growing Rat-1 fibroblasts can be isotopically distinct from the growth medium water. These values drop to ∼25% when the cells have reached quiescence, suggesting that the fluxes of isotopically distinct H and O atoms are derived from metabolic processes.

It is perhaps surprising that mammalian cells maintain such a large isotopic gradient across their membranes after they have reached quiescence and their metabolic activity supposedly declines. Our data, however, indicate that while the fluxes of H and O atoms do indeed decline during quiescence, they do not decrease as much as might be expected, indicating that the cells continue to maintain a reasonable level of metabolic activity. This conclusion is supported by recent results demonstrating that quiescent primary human fibroblasts maintain high activity in a variety of metabolic pathways [20].

Employing FTIR spectromicroscopy, a novel, completely independent, and complementary technique, we provide additional evidence that metabolic processes result in an isotopically distinct flux of H atoms into water that can be measured experimentally. IR spectra of exponentially growing Rat-1 fibroblasts bathed in a ^2^H_2_O buffer display the presence of ^1^H-O-^2^H stretches and bends that grow in intensity over time, presumably as ^1^H atoms from the growth medium are processed and exchange with ^2^H_2_O. Conversely, when these cells are in lag phase, there is essentially no change in intensity of these bands, indicating that this processing of the ^1^H atoms is dependent on the metabolic activity of the cell. This observation is in agreement with previous observations from FTIR spectromicroscopy experiments. Goff et al. observed that after *Chlamydomonas reinhardtii* cells were washed with ^2^H_2_O, O-^1^H water bands increased in intensity over time, consistent with a metabolic flux of ^1^H from substrates into water [14].

It is interesting to compare these current results with those obtained using *Escherichia coli*. Our previous results [9], [10] revealed that in exponentially growing *E. coli* cells cultured in 2X LB medium, approximately ∼53% of the H atoms and ∼70% of the O atoms are isotopically distinct from growth medium water, and that these values drop to ∼23% and ∼27% for H and O atoms, respectively, in stationary-phase cells. These values are remarkably similar to our current results with Rat-1 fibroblasts. This similarity is perhaps especially surprising given the difference between the percent of total extracted water that is composed of intracellular water. With *E. coli* only ∼15% of the extracted water was intracellular water, while in our experiments with Rat-1 fibroblasts that value was over 50%, a discrepancy that can be rationalized at least in part by the larger volume and hence proportionally smaller surface area of mammalian cells.

We have also demonstrated that isotopically distinct fluxes of H and O atoms can be detected in animal tissue. Approximately 12% of the H and O atoms in water extracted from muscle tissue of laboratory rats were isotopically distinct from the total body water extracted from blood, presumably the result of various metabolic processes. The isotope ratios of these fluxes were estimated to be approximately −40‰ and +10‰ for H and O atoms, respectively. Because it is not feasible to perform “wash” experiments with muscle tissue, we do not know the percentage of extracted water that is composed of intracellular water. Although we therefore cannot calculate the percent of H and O atoms in intracellular water that are isotopically distinct from total body water, we can assign 12% as a lower limit. Regardless of the exact value, however, these data confirm the presence of fluxes of H and O atoms in mammalian cell tissue that are isotopically distinct from total body water, and demonstrate that these fluxes can be experimentally measured.

A reasonable question to ask is whether there are potential sources of error that might account for the isotopically distinct populations of H and O atoms observed in both Rat-1 fibroblasts and rat muscle tissue. Evaporation of water from the samples is one trivial explanation that would alter the isotope ratio of extracted water, leading to isotopic enrichment of the remaining water. This, however, would lead to enrichment of all samples, which is not consistent with the data. Conversely, incomplete extraction of water from the samples would lead to observed H and O isotope ratios that are depleted relative to their true values. But again, this scenario is not consistent with our data because the isotopically distinct H and O atoms are not uniformly depleted relative to the growth water. In addition, neither explanation is consistent with the FTIR data that must account for the incorporation of ^1^H into ^2^H_2_O.

Another potential explanation is that atmospheric water was inadvertently added to our samples. However, all extractions were performed in Salt Lake City where the isotope ratio of local water is depleted in ^2^H and ^18^O (approximately -16‰ for O and -120‰ for H) relative to Vienna Standard Mean Ocean Water. Neither of these values is consistent with the ratios of our isotopically distinct H and O atoms. While it is possible that some other chemical might account for the isotopically distinct H and O atoms in all of the samples, the Rat-1 fibroblasts and muscle tissue were collected at different times and stored in vials of different chemical composition. In addition, examination by thermal conversion elemental analysis indicates that this supposed chemical contaminant must either have the same H/O ratio as water, or it must be present in very low abundance. These and other possible explanations were considered by Kreuzer et al. [10], but the most parsimonious explanation is that there is an isotopic gradient between intracellular and extracellular water, and that this gradient is the result of the fluxes of H and O atoms during metabolic processes.

Importantly, Kreuzer et al. [10] obtained independent evidence that the isotopic content of intracellular water in *E. coli* changed over the growth cycle of the culture by measuring the H isotopic content of fatty acids isolated from the cells during mid-log phase and stationary phase. The isotope ratios of chemically identical fatty acids changed from log to stationary phase in a manner consistent with a changing metabolic flux of H atoms, as predicted from the extracted water data.

In summary, previous work has demonstrated conclusively that metabolic fluxes of O and H can be detected in laboratory cultures of *E. coli*. Here we have extended these findings and shown that metabolic fluxes of O and H can also be detected not only in cultured eukaryotic cells, but in muscle tissue of laboratory rats as well.

## Materials and Methods

### Tissue Culture

Rat-1 fibroblast cells [21], [22] (a generous gift from Bruce Magun, Oregon Health & Science University) were cultured at 37°C in Dulbecco Modified Eagle Medium (DMEM) made with water of varying isotopic content plus 10% fetal calf serum in an incubator containing 5% CO_2_. Twelve plates of cells were cultured for each data point. Cells were harvested either during exponential growth (∼30% confluent) or after they had become quiescent (∼100% confluent) via brief trypsinization followed by gravity filtration through a 1.2 µm nylon filter (GE Osmonics #R12SP320F5). The cells were maintained at 37°C during the filtration process. Water was then extracted cryogenically from both the cell pellets and the spent medium samples as previously described [9].

### Muscle Tissue Samples

We raised three groups of male Sprague-Dawley rats (Harlan Teklad) on isotopically different waters to determine if isotopic gradients due to metabolism exist and can be measured in mammals. Water treatments were introduced when animals weighed 35–49 g. Two of the rats were supplied with water that was isotopically depleted in both ^2^H and ^18^O (δ^2^H  =  −120±2‰; δ^18^O  =  −16.0±0.3‰), two were supplied with water that was isotopically enriched in ^2^H and depleted in ^18^O (δ^2^H  = 348±2‰; δ^18^O  =  −15.8±0.2‰), and three were supplied with water that was isotopically enriched in both ^2^H and ^18^O (δ^2^H  = 348±3‰; δ^18^O  = 15.5±0.1‰). Depleted water was Salt Lake City tap water while enriched water was made by adding ^2^H_2_O and/or H_2_^18^O (Cambridge Isotope Laboratories, Inc) to tap water. All rats were maintained on rat chow *ad lib* (Harlan #8640; δ^2^H  =  −109±2‰; δ^18^O  = 25.2±0.3‰) and allowed to mature into adults (∼3 months; ∼340 g).

Rats were euthanized (mean mass at sacrifice: 338.9±21.6 g) with an overdose of Isoflorane and immediately dissected. Blood was extracted from the left ventricle of the heart using a 3-mL syringe and a 22 gauge needle. Lateral gastrocnemius (muscle) slices were prepared by slicing a 1 mm thick piece of tissue with a tool designed to hold two surgical razor blades. Tissue slices were blotted with laboratory wipes, placed in air-tight glass vials, and frozen in dry ice. Water was extracted cryogenically from the samples on the same day they were obtained according to a previously described procedure [9]. All experimental protocols were approved by the University of Utah Institutional Animal Care and Health Committee (#05-08011).

### Stable-Isotope Ratio Measurements

Stable-isotope ratio measurements were made at the Stable-Isotope Ratio Facility for Environmental Research at the University of Utah in Salt Lake City. Stable-isotope ratios are measured relative to internationally recognized standards [23]. We calibrate laboratory standards to the international standards, and then include the laboratory standards as internal standards in every run. Stable isotope contents are expressed in “delta” notation as δ values in parts per thousand (‰), where δ‰  =  ((R_A_/R_Std_) –1) * 1000‰, and R_A_ and R_Std_ are the molar ratios of the rare to abundant isotope (e.g. ^2^H/^1^H) in the sample and the standard, respectively. The standard used for both oxygen and hydrogen is Vienna Standard Mean Ocean Water [23].

The oxygen and hydrogen stable isotope ratios of water samples were determined on a ThermoFinnigan-MAT Delta Plus XL isotope ratio mass spectrometer (IRMS, Bremen, Germany) equipped with a Thermal Conversion Elemental Analyzer (ThermoFinnigan-MAT, Bremen Germany) and a GC-PAL autosampler (CTC Analytics, AG, Zwingen, Switzerland) [24]. The injection volume was 0.5 µL. Water samples were analyzed in duplicate and the results averaged. The average standard deviation of repeated measurements of water standards was ∼2‰ for H isotopes and 0.2‰ for O isotopes.

### Fourier Transform Infrared (FTIR) Spectromicroscopy Measurements

Rat-1 fibroblast cells were generously provided by Prof. Sabine Mai (University of Manitoba) and Prof. Kurt Ballmer (Paul Scherrer Institut). The measurements were repeated using the two different sources of cells in two different laboratories. The cells were seeded on circular (25 mm in diameter and 1 mm thick) CaF_2_ optical windows (Crystran, Dorset, UK) in 30 mm polystyrene tissue culture dishes containing 3 mL of complete DMEM (Invitrogen, Carlsbad, Ca, USA) medium supplemented with 10% Fetal Calf Serum (Invitrogen, Carlsbad, CA, USA) at 37°C in a humid atmosphere of 5% CO_2_.

After the cultures reached the desired degree of confluence (∼60%), the CaF_2_ windows were removed from the incubator and washed with serum-free DMEM medium. The windows were then gently rinsed twice with serum-free DMEM medium prepared in 99% ^2^H_2_O (SIGMA, St. Louis, MO, USA). The windows were transferred to an IR sample holder for solutions and a few drops (10–50 µL) of the same DMEM solution in ^2^H_2_O were added to ensure the presence of a uniform thin aqueous film covering the window. A 15 µm thick circular PTFE spacer was used to limit the optical path through the sample holder and avoid crushing the cells between the two CaF_2_ windows.

The IR holder was inspected under the microscope, and a region containing a cluster of cells of the desired size suitable for the measurement was identified (typically a cluster filling the entire confocal aperture of the microscope). Measurements were performed with the IR microscope confocal apertures set at 25 µm×25 µm. A background measurement was performed in a spot where no cells were present and the stage was then moved to bring the selected cell cluster on the aperture. Spectra were measured repeatedly in the same location every minute for several hours. The spectral changes were plotted either as absorbance spectra calculated relative to the cell-free background or as absorbance variations from the start of the experiment. In a separate control experiment, cell viability was verified by mixing a solution of 0.2% Trypan Blue in ^2^H_2_O in a 1∶1 ratio with the DMEM/^2^H_2_O; the lack of staining of the nuclei indicated that the vast majority of cells were intact.

FTIR measurements were conducted on the endstations of beamline 01B1-01 at the Canadian Light Source and beamline X01DC at the Swiss Light Source, using the internal globar source. The endstations are composed of a Bruker IFS 66v/S interferometer (01B1-01) and a Bruker Vertex70 (X01DC) coupled to a Hyperion 2000 IR microscope (Bruker Optics, Billerica, MA, USA). Light was focused and collected by a 36× Schwarzschild condenser and objectives, and consequently detected by a liquid N_2_-cooled narrowband HgCdTe (MCT) detector. Data was recorded over the mid infrared region using a KBr/Ge multilayer beamsplitter, while scanning at a spectral resolution of 4 cm^−1^. Single channel traces were obtained using the fast Fourier transform algorithm, without zero-filling, after applying a Blackman-Harris 3-Term apodization function. Spectra were collected using 256 scans per spectrum with a 40 kHz acquisition rate.

Data analysis was performed using OPUS version 6.5 (Bruker Optics, Billerica, MA, USA), and data were plotted using Origin version 8.0 (OriginLab, Northampton, MA, USA).
